# Application of Mixed Methods to Identify Small Ruminant Disease Priorities in Ethiopia

**DOI:** 10.3389/fvets.2019.00417

**Published:** 2019-11-26

**Authors:** Biruk Alemu, Hiwot Desta, Wole Kinati, Annet A. Mulema, Solomon Gizaw, Barbara Wieland

**Affiliations:** ^1^Animal and Human Health Research Program, International Livestock Research Institute (ILRI), Addis Ababa, Ethiopia; ^2^International Center for Agricultural Research in the Dry Areas (ICARDA), Addis Ababa, Ethiopia

**Keywords:** participatory epidemiology, small ruminant, diseases, gender, smallholders

## Abstract

Animal health interventions tend to focus on transboundary or zoonotic animal diseases and little attention is given to diseases that mainly affect livestock production and productivity which are of concern for smallholder farmers. To understand disease priorities of men and women livestock keepers and how these impact households, this study used participatory methods to elucidate priorities, reasons for prioritization, knowledge on small ruminant diseases and their transmission pathways. The study was conducted in 23 sites distributed across 14 districts in four regional states of Ethiopia. Ninety-two focus group discussions (FGD) were conducted with men or women only groups. Various tools, such as semi-structured interviews, simple scoring and proportional piling were used to facilitate the process. A follow-up household survey involving 432 households/interviewees collected in-depth data on key small ruminant diseases. Each focus group identified and scored their top five diseases. During analysis, the diseases were grouped in to seven major categories based on local names and clinical signs reported. Highest scores in proportional piling (out of 100 counters) were obtained for respiratory diseases and gastrointestinal parasites in highland areas (mixed crop-livestock systems) with strong agreement among respondent groups using Kendall's coefficient of concordance (*W*) (*W* = 0.395, *p* < 0.01); whereas in lowland areas (pastoral and agro-pastoral systems), the priorities were respiratory and neurological diseases, also with very strong agreement (*W* = 0.995, *P* < 0.01). There was no significant difference between men and women in prioritizing disease constraints. The reasons for prioritization were also used to define categories of impact of disease. The household survey confirmed disease priorities and highlighted the role of mortality for respiratory diseases. Despite differences in household roles, both men and women unvaryingly described the clinical signs in live animals the same way and reported similar observations of disease in carcasses of slaughtered animals. Overall, both men and women farmers had low awareness of zoonotic diseases. In conclusion, the priorities of national disease control programs do not fully match priorities of farmers. Such participatory tools should therefore, play a pivotal role when designing sustainable livestock health interventions.

## Introduction

In Ethiopia, small ruminants serve multiple livelihood functions by providing food and nutrition, income and raw material for industries. They also serve various important cultural purposes such as wedding gift, charity to poorer relatives and inheritance. Women have a lot of control over small ruminants compared to other livestock species and are closely involved in small ruminant health management. The animals often serve as emergency sources of funds for household obligations and personal use. The increase in demand for small ruminant meat products both locally and internationally presents an opportunity for small ruminant keepers to access better markets. However, this opportunity has not been used because of underperformance of the value chain in part attributed to the inability of producers to supply safe products in required quantities.

Infectious diseases have a huge impact on productivity of smallholder livestock systems and repeatedly come up as major constraints in household surveys in Ethiopia (1). The impact of animal diseases is seen through direct losses due to mortality, cost of treatment and indirect effects through slow growth and low fertility linked to morbidity. Annual mortality ranges from 12 to 14% for sheep and 11 to 13% for goats in Ethiopia (2). Animal health research and development projects and government interventions tend to deal with animal diseases which affect trade, are transboundary in nature, or are zoonotic. Comparably, little work has been done on endemic diseases and their contribution to loss of productivity is poorly documented even though these diseases potentially play an important role in adversely affecting food security and the livelihood of smallholder farmers.

To ensure that views of farmers are understood, veterinarians began using participatory methods in the 1980s, particularly in community-based livestock projects in Africa and Asia. By the late 1990s, there was increasing use of these methods and the term “participatory epidemiology” was commonly used to describe veterinary applications of participatory rural appraisal (PRA)-type approaches and methods. While PRA is a multidisciplinary approach to various development problems in rural communities, participatory epidemiology has increasingly been used by veterinarians with a focus on livestock diseases (3).

Besides participatory qualitative research approaches, use of mixed methods, which involves collecting and analyzing qualitative and quantitative data in a single study, have been also successfully applied in animal health (4–7). These approaches can provide a better understanding of the research problem than either approach alone (8).

This gender sensitive study on disease constraints was conducted using a mixed methods design with the aim of generating evidence to design interventions to address key animal health constraints. The study objectives were to identify the main small ruminant disease constraints as perceived by men and women, the impact of the diseases on households, knowledge of men and women on small ruminant diseases, their respective involvement in disease management and understanding disease transmission pathways. The findings will help to influence the national policy on livestock disease surveillance systems and disease control to ensure that smallholders' problems are addressed.

## Materials and Methods

### Study Areas

The study was conducted in 23 villages across 14 districts in Amhara, Oromia, Tigray and Southern Nations Nationalities People's (SNNP) regions of Ethiopia. Five districts in the Amhara region (Basona Worena, Menz Gera, Menz mama, Abergelle and Ziquala), three districts in the Oromia region (Sinana, Yaballo, and Horro), three districts from SNNP (Lemo, Doyogena and Menjiwo/Adiyo), and three districts in the Tigray region (Endemehoni, Atsbi wonberta, and Tanqua Abergelle) participated.

The agroecology and production system characteristics of the study sites are shown in Table 1. Livestock production in Ethiopia is broadly classified into pastoral, agro-pastoral and mixed crop-livestock, peri-urban and urban production systems (9).

**Table 1:** Study sites, agroecology, and production system characteristics.

**Region**	**Districts**	**Sites/villages**	**Agroecology**	**Production systems**	**Altitude (m)**	**Rainfall (mm)**	**Temperature (°C)**
Amhara	Basona Worena	Goshe bado	Moist highland	Mixed crop-livestock	2,419	948	16
	Basona Worena	Gudo beret	Moist highland	Mixed crop-livestock	3,142	1,118	12
	Menz Gera	07 (Yedilfere)	Moist highland	Mixed crop-livestock	3,097	1,261	12
	Menz Mama	06 (Delfanna)	Moist highland	Mixed crop-livestock	3,097	1,261	12
	Abergelle	Sazaba	Dry lowland	Mixed crop-livestock			
	Ziquala	Bilewaqu	Dry lowland	Mixed crop-livestock	1,486	732	22
Oromia	Sinana	Selka Bakaye	Moist highland	Mixed crop-livestock	2,486	1,017	14
	Sinana	Ilu sambitu	Moist highland	Mixed crop-livestock	2,372	1,039	15
	Yabello	Elewaya	Dry lowland	Pastoral/agro-Pastoral	1,181	493	22
	Yabello	Derito	Dry midland	Pastoral/agro-Pastoral	1,588	625	20
	Horro	Lakku iggu	Wet highland	Mixed crop-livestock	2,678	1,621	13
	Horro	Gitilo Dole	Wet highland	Mixed crop-livestock	2,640	1,604	14
SNNPR	Lemo	Jawe	Moist mid-land	Mixed crop-livestock	2,152	1,136	17
	Lemo	Upper Gana	Moist mid-land	Mixed crop-livestock	2,151	1,086	17
	Doyogena	Ancha Sadicho	Moist highland	Mixed crop-livestock	2,616	1,314	14
	Doyogena	Hawara Arara	Moist highland	Mixed crop-livestock	2,499	1,275	15
	Adiyo	Boka	Wet highland	Mixed crop-livestock	2,464	1,910	15
	Adiyo	Shuta	Wet highland	Mixed crop-livestock	2,316	1,871	15
Tigray	Endemehoni	Embahasti	Dry highland	Mixed crop-livestock	2,884	746	14
	Endemehoni	Tsebet	Dry highland	Mixed crop-livestock	3,184	796	13
	Atsbi wonberta	Golgol Naele	Dry highland	Mixed crop-livestock	2,691	608	15
	Atsbi wonberta	Habes	Dry highland	Mixed crop-livestock	2,559	588	16
	Tanqua Abergelle	Hadnet/Hibiret	Dry lowland	Mixed crop-livestock	1,442	653	22

The highland agroecology with mixed crop-livestock system is typical for areas above 2,200 m above sea level (masl) and is characterized as a system in which livestock husbandry and rain fed cropping are closely interlinked. Livestock provide inputs (draft power, transport, manure) to other parts of the farm system and generate consumable or saleable outputs (milk, meat, eggs, hides and skins, wool, hair and manure). Crop residues are used as livestock feed; animals can be sold and revenues reinvested in agriculture or sold when the crop is failing because of weather or pests; cereals and most staple foods are produced in quantities that cover the needs of the family and excess is sold. The principal objective of farmers engaged in mixed farming is to gain complementary benefit from an optimum mixture of crop and livestock and spreading income and risks over both crop and livestock production (10).

The lowland agroecology with mixed crop-livestock system denotes elevation ≤1,500 masl where farmers herd livestock in rangelands and produce crops on fertile land. The system is understood in dual sense: firstly, it refers to farming systems entirely based on livestock but practiced in proximity to and perhaps functional association with cropping farming systems; secondly, it refers to the livestock subsystem of crop-livestock farming.

The lowland agroecology is typical for the pastoral production system characterized by sparsely populated pastoral rangelands, where subsistence of pastoralists is mainly based on livestock and livestock products. Livestock husbandry in this system is dominated by goats, cattle, sheep, and camels. Since the main source of food is milk, pastoralists tend to keep large herds to ensure sufficient milk supply and generate income by selling dairy products or live animals. The pastoral production system in some areas has been evolving into an agro-pastoral system (9). The agro-pastoral form of livestock production dominates in mid agroecological zones where a tendency for crop production over livestock production has been observed. Agro-pastoralists are sedentary farmers who grow crops and raise livestock. Livestock are used for draft, savings, and milk production. The production system is subsistence type of milk and or meat production (11). Cattle and small stock play a critical role in the agro-pastoralist household economy.

### Ethics Approval Statement

This study was carried out in accordance with the recommendations of the ILRI Institutional Research Ethics Committee (ILRI IREC). ILRI IREC is accredited by the National Commission for Science, Technology and Innovation (NACOSTI) in Kenya. The protocol was approved by the ILRI IREC (Certificate Ref. No: ILRI-IREC2015-17). The respondents provided consent to participate in the study by completing the questionnaires.

### Methodology

We used an exploratory sequential type of mixed methods design, using both the qualitative and quantitative approaches equally (QUAL → QUAN) (8, 12, 13). The qualitative data collection preceded the quantitative data collection. The intent was to first explore perceptions of smallholder men and women farmers on priority small ruminant diseases and then follow up on this exploration with quantitative data collection that allows studying a larger sample so to be able to infer results to the targeted population.

**Figure 1:**
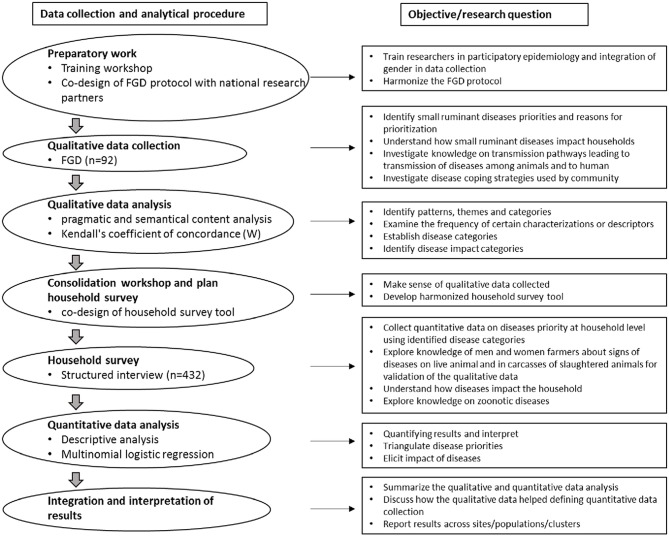
The conceptual overview of mixed methods and analytical approach.

Qualitative data were first gathered through participatory focus group discussions. Quantitative data were then collected through a cross sectional study based on structured interviews for further detail exploration and in order to confirm initial findings. The qualitative part helped to identify emerging questions to be tested, allowed us to define disease categories and impact categories, and also ensured that the quantitative instruments were relevant and adequate. The conceptual overview of the mixed methods and analytical approach used are outlined in Figure 1.

#### Focused Group Discussion

##### Gender relevant framework

For the design of this study, it was hypothesized that men, women, boys, and girls have different knowledge and skills on livestock health depending on their roles and responsibilities in animal husbandry. However, very few studies have explored gender roles and views for small ruminant production in Ethiopia. Following the “gender relevant framework,” which proposed to speaking separately to men and women (14), we paid great attention to capture the views of men and women by conducting separate FGDs and by ensuring women-headed households were included in the household survey. This methodologically relevant frameworks assumes that if interviewed separately, men, and especially women, are more likely to respond openly. Data collected in this way, can be analyzed to understand how to design, deliver and monitor livestock interventions to meet the needs and realities of both men and women (15, 16).

A workshop was held for veterinarians and researchers from national agricultural research institutes to train them in participatory epidemiology and gender. Participants learnt about the use of participatory epidemiology tools for studies on animal diseases problems, and the training had a strong focus on gender, its implication on study design and implementation of research. As part of the workshop, the study protocol was developed and tested in role play, and details of the field work were planned.

Four research teams, each consisting of 4–5 researchers, conducted the surveys.

Before each FGD, a meeting was held with local administration officials and site coordinators to introduce and explain the objectives of the study emphasizing the relevance of disease in small ruminants and their impact on household members. Site coordinators with local knowledge facilitated contact with development agents and farmers. Each team comprised a facilitator and note taker responsible for recording information. As part of the preparation, suitable locations to conduct the FGDs were identified. In each village, four FGDs took place with separate FGDs for men, women, male youth and female youth and a total of 92 FGD were conducted across the study villages. Each FGD had 6–8 participants who were actively involved in small ruminant production and had their own small ruminant herd. Attempts were made to ensure that the men FGDs comprised at least 1–2 local elders and a traditional healer.

The FGDs for men and women were held in parallel and findings of each were briefly presented in a joint session at the end of the FGDs. Similarly, the FGD for young male and female small ruminant keepers were held in parallel and their findings shared at the end during feedback sessions. Key informants (para-veterinarians and community animal health workers) were interviewed for triangulation purposes and to collect additional information or to clarify issues that may have come up during FGDs with farmers.

Various participatory tools were used to facilitate discussions, including simple ranking and proportional piling. First, participants listed five important small ruminant diseases that affect their herds and described the clinical signs of these diseases (local disease names were noted). For the ranking of diseases, 100 beans/counters were distributed among the diseases to indicate their relative importance. In the process, participants were asked to explain the reasons for assigning the scores. The impact of these diseases on households were also identified. For the top two diseases, participants were asked to explain how the disease is spreading between animals, if it can transmit between animals and people, and who of the household is involved in activities that may lead to disease transmission. Ways of transmission or transmission situations were listed. As a last question, participants were asked to explain what the community does in response to small ruminant diseases to understand their disease coping strategies.

#### Household Survey

The findings of the FGDs informed the design of a household survey involving 432 households in the same communities. The FGD participants were excluded from the household survey. This survey aimed to gather more quantitative data to assess disease priorities, knowledge of men and women farmers about small ruminant diseases, the impact of diseases and investigate perceptions of different household members on their involvement in small ruminant health management.

Stratified multistage sampling (17), with four hierarchical stages, was used as sampling strategy. The regions, districts and villages were sampled purposively to include different agro-ecological zones and production systems. The first level of selection was the regions, within each of the selected regions, specific districts and villages were also selected. Households within each study villages were then selected by a stratified sampling approach targeting men-headed households and women-headed households.

To determine the sample size required for the cross-sectional household survey, the sample size and power calculation tool of Epi InfoTM 7 (CDC, Atlanta, GA) was used. The required sample size of 423 was calculated allowing us to be 95% confident of detecting a certain household characteristic or activity if it was practiced by 50% of the households, assuming allowable error of 5% and account for a design effect of 1.1 for clustering at village level. A sampling frame of all households having small ruminants from each of the selected villages was obtained from the respective administration office. In each of the 23 study site/villages, 15 men-headed households and 4 women-headed households were selected using systematic random sampling and then a few extra were added to account for eventual drop-outs, and finally the survey was conducted in 432 households.

Similar to the planning of the participatory survey, the study protocol was developed during training with researchers involved in data collection to ensure harmonization of data collection. For disease priorities, respondents were asked to indicate the top three small ruminant diseases. Impact categories of diseases were derived from answers received in the participatory survey. Knowledge of clinical signs of small ruminant disease in live animals and observation of diseases in carcasses of slaughtered animals was elicited. Information on zoonotic disease were also recorded by asking participants about the type of diseases they know that are transmitted from animals to people and their clinical signs.

### Data Analysis

Epi info software version 7 was used for data collection and entry and exported into a Microsoft Excel 2013 spreadsheet. Data was analyzed using SPSS version 23.

Although the clinical signs described for most of the diseases mentioned by farmers are consistent with clinical signs and indicators described in veterinary literature and textbooks (18, 19), and were crosschecked with key informants, a conclusive diagnosis based on clinical signs and without laboratory confirmation is difficult, unscientific and unreliable. Thus, instead of using specific disease names, the diseases were grouped into seven disease categories.

For the FGD data, pragmatic and semantical content analysis was employed (20). For the pragmatic content analysis, diseases were grouped into categories according to their probable signs and attributed effects as described by the focus groups. Semantical content analysis used attribution analysis to examine the frequency with which certain characterizations or descriptors were mentioned. Descriptive statistics were computed for frequency and the percentage of FGD groups who described different disease priorities, reasons for prioritizing them and disease coping strategies.

The level of agreement among the scores of informant groups (men and women) for priority diseases in different agroecologies and production systems was assessed using Kendall's coefficient of concordance (*W*) (21). Consequently, evidence of agreement between informant groups was categorized as “weak,” “moderate,” and “strong” according to published guidelines on the interpretation of *W* and *P*-values assigned. Agreement was termed “weak” for *W* < 0.26 and *P* > 0.05; “moderate” for *W* = 0.26–0.38 and *P* < 0.05; and “strong” for *W* > 0.38 and *P* < 0.01.

Descriptive statistics were computed with frequency and percentage on the household data to understand knowledge of farmers on diseases.

For both the household data and data from FGDs on disease categories, multinomial logistic regression analysis was performed (17, 22, 23). The seven disease categories were considered as nominal dependent variables in the model. Each of the listed prioritized diseases with their corresponding explanatory variables were treated as one observation, leading to an inclusion of multiple observations per FG/household in the regression. The potential clustering effect was, however, not adjusted because a random effect multinomial regression model cannot be implemented in SPSS. We believe the effect of clustering was minimal because of the small number of observations per FG/household. The perception and preference of farmers were measured by proportional piling, resp. the number of beans allocated to each of the categories of the dependent variable. Reasons behind the scores (impacts of diseases), agroecology and production system, and gender were considered as independent variables. Multicollinearity was checked with simple correlations among the independent variables. Pairwise correlation coefficient > 0.8 considered as evidence of collinearity. Variables with a *p* < 0.2 in the univariable analysis were included in the multivariable analysis. A backward stepwise model building process was employed. The significance of predictors was assessed using a likelihood ratio test and some variables were excluded from the final model because these did not improve the model prediction based on the likelihood ratio test with a *p*-value for variable removal of 0.157 as suggested (17).

The likelihood ratio chi-square test parameter was used to assess if the model predicts significantly better, or more accurately, than the null model. A *p*-value of <0.05 was considered to suggest model fit in the likelihood ratio test. The goodness-of-fit was also assessed through the Pearson chi-square tests, with *p* > 0.05 signifying better fit (24).

In the model (17), for an outcome variable that has *J* categories, the probability of membership in each of the outcome categories was computed by simultaneously fitting *J*-1 separate logistic model (with one category serving as the baseline or reference category). Consequently, for the dependent variable with 7 levels (leaving the first level as the baseline category), we estimated 6 sets of coefficients [β^(2)^, β^(3)^, β^(4)^, β^(5)^, β^(6)^] corresponding to the remaining outcome categories. Because β^(1)^ = 0, the predicted probability that an observation is in category 1 was:

(1) $$\begin{array}{r} P\;\left(y=1\right)=1/1+exp\left(x\beta^{\left(2\right)}\right)+exp\left(x\beta^{\left(3\right)}\right)+\ldots \ldots ..+exp\;\left(x\beta^{\left(7\right)}\right) \end{array}$$

while the probability of being in category 2 was:

(2) $$\begin{array}{r} P\;\left(y=2\right)=exp\left(x\beta^{\left(2\right)}\right)/1+exp\left(x\beta^{\left(2\right)}\right)+exp\left(x\beta^{\left(3\right)}\right)+\ldots \ldots \\ \;+exp\;\left(x\beta^{\left(7\right)}\right) \end{array}$$

and similar for categories 3, 4, 5, 6, and 7.

To identify any difference in the disease priorities of the men and women groups, the multinomial regression was applied separately for the female and male FGD data. The top three diseases categorized in to the seven disease categories were considered as a nominal dependent variable. The farmers' preferences and perceptions measured as the number of beans/scores allocated to each of the categories of the dependent variables was an independent continuous variable (covariate) in the model.

## Results

### Priority Diseases and Reasons for Ranking

Farmers identified several diseases and syndromes that affect small ruminants with local names (vernacular names) and described their clinical signs. Based on this information and in consultation with key informants, the diseases described by participants of FGDs were grouped into seven major disease categories according to their clinical manifestation. These manifestations were respiratory, neurological, skin, gastrointestinal tract (GIT), external parasites, and systemic diseases. Diseases that did not fit into any of these categories were grouped as “others.” Some of the reported diseases were difficult to name scientifically, therefore the descriptions of clinical signs and postmortem lesions mentioned were used to classify them into the respective category (Table 2). The probable corresponding scientific name of these diseases was added based on the clinical manifestations described by the informants. The vernacular names of diseases varied across study regions depending on the local language, even with small variations between districts.

**Table 2:** Disease categories, descriptions or diseases mentioned in the FGDs.

**Major disease categories**	**Description**	**Local names of diseases mentioned**	**Probable scientific name**	**Count of FGDs**	**% of FGD**	**% within the category**
Respiratory disease	Diseases of the respiratory tract: common clinical signs like coughing, sneezing, nasal discharge, dyspnea, abnormal respiratory sounds and different lung lesions at slaughter	Sombeessa^b^/Bubbutaa caccabsaa^b^/Argansoo^b^	Contagious Caprine Pleuropneumonia	13	14.13	11.21
		Sale^a^	Coughing	11	11.96	9.48
		Fuun Duuda^b^	–	1	1.09	0.86
		Engib^a^/Wotewut^a^/Furroo^b^/ Surridoo^b^/Halkafean^c^ /Tegta^c^/Mieta^c^ /Ganshu^d^/Gunfan^d^/Oshiyo^d^	Pasteurellosis	62	68.48	54.31
		Hudhaa^b^/Kokkee^b^	–	1	1.09	0.86
		Qedefera^d^	–	2	2.17	1.72
		Qeli Nefo^d^	–	4	4.35	3.45
		Samba mich^a^/Qufaa^b^/	Pneumonia	20	21.74	17.24
		Sillisaa^b^	–	1	1.09	0.86
Neurological disease	Diseases of the central nervous system: signs of circling, convulsion, staggering, abnormal behavior, abnormal gait and ataxia	Baria wez^a^/Azurit^a^/Tinan^a^/Sirgoo^b^/Lafan Martoo^b^/Jaanjjoo^b^/ Zarti^c^/Hsake Resi^c^/kenin^c^/Azar^c^/Aqnine^c^/Boko hucha^d^/Qele Gudo^d^	Coenurosis	53	57.61	94.64
		Gurgurit^c^/Haseka Riesi^c^	Oestrus ovis	1	1.09	1.79
		Riqannoota^b^	–	2	2.17	3.57
Skin disease	Skin diseases: different skin lesions and signs like hair loss, crusts, scabs, irritation, itching	Fentata^a^/Finnoo^b^/Darrabo^b^/ Shihure^c^/Bededo^c^/ Shilimat^c^/Enfrir^c^	Sheep and goat pox	35	38.04	60.34
		Afemended^a^/Abdarraa^b^/ Dorrobboo^b^/Umburura^b^ Af'tetem^c^/Afe'mear^c^/Kurkursa^d^	Orf	21	22.83	36.21
		Gogimos^d^/Qodi Mosu^d^	–	2	2.17	3.45
Gasto-intestinal tract (GIT) disease	Diseases of the gastrointestinal tract: diarrhea, emaciation, erected hair, ascites, bottle jaw, and presence of different stages of parasite in feces and in GIT at slaughter	Albaatii^b^	–	2	2.17	2.08
		Mawule^a^/Dodo'o^b^/Malullaa^b^/Jiitoo^b^ /Efeel^c^/Haseka Kebdi^c^	Fasciolosis	29	31.52	30.21
		Tsihtsah^c^/Gurgurit^c^/ Hamasu^d^/Temu^d^	GIT Parasites	8	8.70	8.33
		Himam sunba^c^	Lung warm	6	6.52	6.25
		Macho achi cheno^d^	Ascites	6	6.52	6.25
		Malula^d^/Lomme'eta^d^/wochiwocha^d^	Bottle jaw	10	10.87	10.42
		Mototo^d^/Qete nafo^d^	–	1	1.09	1.04
		Teqmate^a^/Albaasaa^b^/Zeaso^d^/Adora^d^	Diarrhea	34	36.96	35.42
External parasite	External parasites resulting in alopecia, itching, irritation, disturbance while grazing, visible to naked eye, externally on the skin of animals (tick, lice, sheep ked)	Ekek^a^/Cittoo^b^/Abeq^c^	Mange mite	19	20.65	55.88
		Aliqaru^d^/Bacharuta^d^	Lice infestation	3	3.26	8.82
		Mezger^a^/Benqera^d^	Tick infestation	11	11.96	32.35
		Qurdid^c^	Sheep ked	1	1.09	2.94
Systemic disease	Multi-systemic diseases with a range of clinical signs, incl. acute severe syndromes	Dhukkuba Tiruu^b^	Necrotic hepatitis	8	8.70	14.55
		Entit^a^/Hamot kebdi^c^/Minkae^c^/Tafia^c^/Megerem^c^/ kenkento^d^/	Anthrax	21	23.91	40.00
		Marra-Reeba^b^	–	3	3.26	5.45
		Qandhoo^b^	General septicemia	6	6.52	10.91
		Sheleme^a^/Tsehtsah^c^/weqie^c^	PPR	12	13.04	21.82
		Dira^a^/Werchi^c^	Black leg	4	4.35	7.27
Others	Diseases with unknown cause, non-infectious, metabolic in nature and difficult to be classified in the above listed categories	Yehode menefat^d^	Bloating	1	1.09	2.38
		Dengetegna Besheta^a^	Sudden disease	1	1.09	2.38
		Dhukkuba hinbeekkamne^b^/Maqaan hin beekamu^b^	Unknown Disease	6	6.52	14.29
		Ayin Beshita^a^/Elemoosu^d^/Elitiso^d^	Eye disease	6	6.52	14.29
		Ye egir til^a^/Maasaa^b^/Barga'oo^b^/Ho'ichoo^b^/ Nutero^d^/ Loka Hucha^d^/ loka shokota^d^/ Moyale^d^/Naqarisa^d^	Foot rot	18	19.57	42.86
		Himam tub^c^	Mastitis	1	1.09	2.38
		Koyooo^d^/wugati^d^	–	1	1.09	2.38
		Mbray^c^	Abortion	1	1.09	2.38
		Mecho^d^/mechi^d^	–	5	5.43	11.90
		Raammoo miilaa^b^	–	1	1.09	2.38
		Senbecha/chinyako^d^	–	1	1.09	2.38

a *Amhara*.

b *Oromia*.

c *Tigray*.

d *SNNP*.

Ten specific diseases or disease syndromes were mentioned by at least 15% of the FGD groups among their top five most important diseases (Table 2). These were pasteurellosis, pneumonia, coenurusis, sheep and goat pox, orf, fasciolosis, diarrhea, mange mite infestation, anthrax, and foot rot.

Some diseases were reported across agroecologies and production systems, while others were particular to an agroecology and production system in one or two districts. For example, pasteurellosis and coenurosis were repeatedly mentioned across production systems and agroecologies with different local names by 68 and 57% of focus groups, respectively. Peste des petits ruminants; however, was mentioned only in lowland mixed crop-livestock production systems (13% of focus groups).

There were important differences when disaggregating data based on the agroecology and production systems of the study districts. Respiratory diseases ranked first in the highland agroecology and mixed crop-livestock production system, followed by GIT diseases and neurological diseases with strong agreements among the informant groups (*W* = 0.395, *P* = 0.000). The most frequently mentioned diseases within the respiratory disease category were pasteurellosis, pneumonia, and contagious caprine pleuropneumonia (CCPP) among 54, 17, and 11% of focus groups, respectively. Diarrhea and fasciolosis were the priority health problems mentioned in the GIT disease category (Table 2).

In the lowland mixed crop-livestock system, the ranking was different with systemic diseases followed by GIT and skin diseases considered priorities with strong agreement among the focus groups (*W* = 0.442, *P* = 0.000) (Table 3). Anthrax and PPR were the major diseases mentioned in the systemic disease category among 40 and 21% of focus groups, respectively. For skin diseases, sheep and goat pox was mentioned by 60% of the focus groups, followed by Orf with 36%.

**Table 3:** Summary of ranking of priority disease categories in different agroecology and production systems (top ranked disease in each FGD got a score of 7).

	**Highland mixed crop-livestock**		**Lowland mixed crop-livestock**		**Midland mixed crop-livestock**		**Lowland pastoral and agro-pastoral**		**Midland pastoral and agro-pastoral**	
**Disease category**	**Mean rank score**	**Test statistics**	**Mean rank score**	**Test statistics**	**Mean rank score**	**Test statistics**	**Mean rank score**	**Test statistics**	**Mean rank score**	**Test statistics**
Respiratory	6.07	*N* = 64 Kendall's *W* = 0.395 χ^2^ = 151.7 Df = 6 Sig. = 0.000	3.63	*N* = 12 Kendall's *W* = 0.442 χ^2^ = 31.83 Df = 6 Sig. = 0.000	4.75	*N* = 8 Kendall's *W* = 0.389 χ^2^ = 18.68 Df = 6 Sig. = 0.005	6.50	*N* = 4 Kendall's *W* = 0.955 χ^2^ = 22.93 Df = 6 Sig. = 0.001	6.00	*N* = 4 Kendall's *W* = 0.847 χ^2^ = 20.33 Df = 6 Sig. = 0.002
Neurological	4.27		2.92		1.81		6.50		7.00	
Skin	4.03		4.46		4.38		2.38		2.75	
GIT	5.18		4.46		5.50		2.38		2.25	
Ectoparasites	2.70		3.67		4.44		2.88		2.25	
Systemic	2.58		6.58		2.56		5.00		4.00	
Other	3.17		2.29		4.56		2.38		3.75	

In pastoral/agro-pastoral production systems in the lowlands and midlands, respiratory diseases and neurological diseases were most important, followed by systemic diseases, again with very strong agreements among the informant groups (*W* = 0.995, *P* = 0.001). Coenurosis (95%) was the major disease stated in the neurological disease category (Table 3).

The main reasons for allocating the scores during the FGDs are presented in Table 4. For example, 56.8% of FGDs mentioned high mortality as the reason for scoring respiratory disease category highest. The main reason described by the farmers for prioritizing neurological diseases was the fact that there is no treatment available.

**Table 4:** Percentage of farmers providing specific reasons for ranking diseases from 1st to 3rd.

**Reasons for ranking**	**Disease category**	**1st rank**		**2nd rank**		**3rd rank**	
		**%of total FGD**	**% within the disease category**	**% of total FGD**	**% within the disease category**	**% of total FGD**	**% within the disease category**
High mortality	Respiratory diseases	22.8	56.8	14.1	39.4	5.4	27.8
	Neurological diseases	2.2	11.8	4.3	36.4	0.0	0.0
	Skin disease	3.3	37.5	3.3	27.3	0.0	0.0
	GIT diseases	10.9	66.7	4.3	22.2	9.8	39.1
	External parasites	0.0	0.0	0.0	0.0	0.0	0.0
	Systemic diseases	6.5	75	3.3	37.5	6.5	37.5
	Others	2.2	50	1.1	11.1	1.1	11.1
Acute and fatal	Respiratory diseases	7.6	18.9	3.3	9.1	1.1	5.6
	Neurological diseases	1.1	5.9	0.0	0.0	0.0	0.0
	Skin disease	0.0	0.0	0.0	0.0	0.0	0.0
	GIT diseases	1.1	6.7	0.0	0.0	0.0	0.0
	External parasites	0.0	0.0	0.0	0.0	0.0	0.0
	Systemic diseases	6.5	75	0.0	0.0	2.2	12.5
	Others	1.1	25	4.3	44.4	1.1	11.1
Unmarketable skin	Respiratory diseases	0.0	0.0	0.0	0.0	1.1	5.6
	Neurological diseases	0.0	0.0	0.0	0.0	0.0	0.0
	Skin disease	4.3	50	4.3	36.4	0.0	0.0
	GIT diseases	0.0	0.0	1.1	5.6	0.0	0.0
	External parasites	1.1	33.3	0.0	0.0	2.2	33.3
	Systemic diseases	0.0	0.0	0.0	0.0	0.0	0.0
	Others	1.1	1.1	0.0	0.0	0.0	0.0
Render the meat inedible	Respiratory diseases	4.3	10.8	0.0	0.0	1.1	5.6
	Neurological diseases	0.0	0.0	0.0	0.0	0.0	0.0
	Skin disease	4.3	50	3.3	27.3	0.0	0.0
	GIT diseases	1.1	6.7	2.2	11.1	0.0	0.0
	External parasites	0.0	0.0	0.0	0.0	1.1	16.7
	Systemic diseases	1.1	12.5	0.0	0.0	0.0	0.0
	Others	0.0	0.0	0.0	0.0	0.0	0.0
High transmission rate/affect most of the flock	Respiratory diseases	21.7	54.1	14.1	39.4	6.5	33.3
	Neurological diseases	1.1	5.9	2.2	18.2	0.0	0.0
	Skin disease	6.5	75	7.6	63.6	6.5	50
	GIT diseases	4.3	26.7	3.3	16.7	2.2	8.7
	External parasites	1.1	33.3	0.0	0.0	3.3	50
	Systemic diseases	3.3	37.5	3.3	37.5	0.0	0.0
	Others	1.1	25	4.3	44.4	1.1	11.1
Frequent occurrence/seen throughout the whole season/endemicity	Respiratory diseases	6.5	16.2	7.6	21.2	3.3	16.7
	Neurological diseases	5.4	29.4	0.0	0.0	0.0	0.0
	Skin disease	1.1	12.5	2.2	18.2	3.3	25
	GIT diseases	12	73.3	7.6	38.9	4.3	17.4
	External parasites	2.2	66.7	0.0	0.0	0.0	0.0
	Systemic diseases	1.1	12.5	2.2	25	0.0	0.0
	Others	1.1	25	2.2	22.2	0.0	0.0
No treatment and recovery	Respiratory diseases	4.3	10.8	2.2	6.1	0.0	0.0
	Neurological diseases	13	70.6	5.4	45.5	8.7	100
	Skin disease	0.0	0.0	0.0	0.0	0.0	0.0
	GIT diseases	2.2	13.3	0.0	0.0	0.0	0.0
	External parasites	0.0	0.0	0.0	10.0	0.0	0.0
	Systemic diseases	0.0	0.0	1.1	12.5	1.1	6.3
	Others	1.1	25	2.2	22.2	0.0	0.0
Market price devaluation/affect market	Respiratory diseases	0.0	0.0	4.3	12.1	0.0	0.0
	Neurological diseases	2.2	11.8	1.1	9.1	0.0	0.0
	Skin disease	0.0	0.0	0.0	0.0	0.0	0.0
	GIT diseases	1.1	6.7	0.0	0.0	0.0	0.0
	External parasites	0.0	0.0	0.0	0.0	0.0	0.0
	Systemic diseases	0.0	0.0	0.0	0.0	0.0	0.0
	Others	0.0	0.0	0.0	0.0	0.0	0.0
High morbidity/affect productivity/reduce body weight/ lead to abortion	Respiratory diseases	0.0	0.0	3.3	9.1	3.3	16.7
	Neurological diseases	1.1	5.9	1.1	9.1	0.0	0.0
	Skin disease	1.1	12.5	3.3	27.3	2.2	16.7
	GIT diseases	1.1	6.7	5.4	27.8	6.5	26.1
	External parasites	0.0	0.0	1.1	50	1.1	16.7
	Systemic diseases	1,1	12.5	1.1	12.5	0.0	0.0
	Others	0.0	0.0	2.2	22.2	3.3	33.3
Not common in the locality	Respiratory diseases	0.0	0.0	2.2	6.1	2.2	11.1
	Neurological diseases	0.0	0.0	0.0	0.0	0.0	0.0
	Skin disease	0.0	0.0	1.1	9.1	5.4	41.7
	GIT diseases	0.0	0.0	3.3	16.7	5.4	21.7
	External parasites	0.0	0.0	0.0	0.0	0.0	0.0
	Systemic diseases	0.0	0.0	0.0	0.0	10.9	62.5
	Others	0.0	0.0	0.0	0.0	4.3	44.4

The reasons mentioned by the farmers implied the impact of the disease. Interesting to note is the fact that none of the focus groups mentioned impact on human health as a reason for prioritizing diseases. Farmers described economic impact as the loss of income from sale of animals and hides. Fifty focus groups stated that they cannot sell hides from infected animals if the animals were affected by skin disease like sheep and goat pox. Most of the time, they also cannot slaughter infected animals for consumption or sale in market. Diseases like PPR, anthrax and pasteurellosis can cause high loss because of high mortality rates. This aggravates poverty for those whose livelihoods depend on these animals. Important to note is also that 73.3% of the focus groups which prioritized GIT diseases mentioned frequent occurrence or endemicity as a reason for scoring them high.

The odds of allocating higher scores to respiratory and GIT disease categories than the reference “other” category were 1.10 (10%) and 1.08 (8%) times for men and 1.14 (14%) and 1.07 (7%) for women focus group participants, respectively. Women and men also gave similar scores to neurological diseases OR = 1.04 (4%) than to the reference “other” category, but the result was significant for women (*p* < 0.05) unlike the men (Table 5). The scoring of diseases provided by the women FGD groups was similar to the scoring by the men groups, this showed the perceptions and priorities of the men and women groups were very similar.

**Table 5:** The odds of farmers allocating different scores to disease categories by male and female focus groups.

**DC^a^**	**Male focus groups**				**Female focus groups**				
	**B**	**Std. error**	**Sig. (P)**	**Odds ratio**	**B**	**Std. error**	**Sig. (P)**	**Odds ratio**	
Respiratory	Intercept	−1.350	0.489	0.006		−2.040	0.429	0.000	
	Score	0.098	0.028	0.000	1.103	0.127	0.021	0.000	1.135
Neurological	Intercept	−0.466	0.397	0.240		−0.419	0.282	0.137	
	Score	0.048	0.027	0.080	1.049	0.043	0.019	0.027	1.044
Skin	Intercept	−0.275	0.381	0.471		−0.306	0.275	0.265	
	Score	0.031	0.028	0.256	1.032	0.033	0.019	0.087	1.034
GIT	Intercept	−0.859	0.433	0.048		−0.796	0.308	0.010	
	Score	0.073	0.027	0.006	1.076	0.068	0.019	0.000	1.071
Ectoparasites	Intercept	0.028	0.360	0.938		0.241	0.248	0.330	
	Score	−0.004	0.030	0.892	0.996	−0.044	0.025	0.076	0.957
Systemic	Intercept	−0.072	0.366	0.843		0.011	0.258	0.966	
	Score	0.010	0.029	0.739	1.010	−0.002	0.021	0.942	0.998

a *The reference category is “others”; NE, non estimable*.

### Impact of Small Ruminant Diseases on Households

The FGDs helped to define categories of how small ruminant diseases impact farmers in terms of financial loss that arises from mortality, lower productivity, loss of marketing value, and treatment costs. It also affects human health including through malnutrition, migration in search of other jobs, children drop out of school because of the extra time needed to take care of sick animals and other related financial constraints; all these causing severe social and psychological impact.

Farmers described financial loss as the loss of income from sale of animals and hides. They also can't slaughter infected animals for consumption or sale in market or sell hides from infected animals. Participant farmers said that it is not even possible to use hides as rugs at home if the animals were affected by diseases like sheep and goat pox.

Some of the diseases are treatable but farmers explained that expenses incurred because of treatment affects the economy of the household. Diseases can also cause loss because of high mortality rates. This aggravates poverty for those whose livelihood is dependent on these animals. Some respondents reported that they were not able to send children to school anymore. It also predisposes household members to migrate to towns or other areas in search of other work.

Farmers also explained that lambs and kids cannot get enough milk from diseased dams, which may lead to death and thus reduce the flock size and greatly affect the overall farm productivity. In some areas where goat milk is consumed, respondents mentioned that there is a drop in milk supply from diseased animals affecting the nutrition of their children.

Also mentioned was the psychological impact that results from losing animals to diseases because in most of the surveyed rural areas ownership of animals is an indicator of wealth determining one's social status. Once a disease enters their sheep and goat flock it makes them give up on rearing other livestock as well.

Adverse social impact was described as being unable to pay taxes, buy fertilizer or pay membership fees to their “Idir” (traditional financial association). In addition, a household which encounters a disease in its flock first will not be allowed by neighbors to mix its herd on grazing fields and watering areas. This sometimes leads to social conflicts.

### Household Survey: Disease Priorities and Impact

The household survey asked for the top three diseases at household level and how these diseases impact the household. The priority diseases mentioned were not different from those in the FGDs (Table 6). Based on results from the FGDs, categories of how of small ruminant diseases impact farmers were defined—economic/income loss, mortality, loss of productivity, loss of value, treatment costs, migration for other jobs, wastage of time treating the animals, children drop out of school, malnutrition, social and psychological impact. In addition, human health was added as a possible impact category.

**Table 6:** Percentage of farmers providing 1st and 2nd priority diseases in different agroecology and production systems.

	**Highland mixed crop-livestock**		**Lowland mixed crop-livestock**		**Mid-land mixed crop-livestock**		**Lowland pastoral and agro-pastoral**		**Mid-land pastoral and agro-pastoral**	
**Diseases**	**1st priority (%)**	**2nd priority (%)**	**1st priority (%)**	**2nd priority (%)**	**1st priority (%)**	**2nd priority (%)**	**1st priority (%)**	**2nd priority (%)**	**1st priority (%)**	**2nd priority (%)**
Pasteurellosis	32.4	14.5	12.5	16.4	27.8	25				
Coughing	18.1	14.5			11.1					
Coenurosis	20.6	21.2					12.5	41.7	8.3	25
Sheep and goat pox	3.9	4.5		31.1						
Liver fluke	4.3	9.3			8.3					
Anthrax			47.2	21.3						
PPR			16.7	11.5						
Diarrhea		12.6	16.7	18		16.7				
CCPP							70.8		75	16.7
GIT disease							16.7	12.5		
Orf					19.4					
Foot rot					16.7	11.1				
Necrotic hepatitis								16.7	16.7	50
Tick infestation						19.4				

The multinomial logistic regression results shown in Table 7 revealed the odds of mentioning the given impact for a given disease category compared to the reference category. Respiratory diseases like CCPP and pasteurellosis had significantly higher odds for high scores due to associated high mortality compared to all other disease categories except systemic diseases (PPR, anthrax etc.). For example, neurological diseases (Odds Ratio, OR = 0.23), skin diseases (OR = 0.47), gastrointestinal diseases (OR = 0.3), and ectoparasites (OR = 0.04) have got significantly lower scores than respiratory diseases due to the high mortality impact associated with respiratory diseases. Systemic diseases (OR = 6.08) compared to respiratory diseases were more likely to affect the lowland mixed crop-livestock production system than midland pastoral and agro-pastoral production system. Other important significant findings were that the amount of time it took to treat diseases was an important reason for scoring “other diseases” high. Diseases mentioned there were foot rot, mastitis, abortion, eye disease, and bloating.

**Table 7:** Multinomial logistic regression coefficients and odds ratios of disease impacts for the top two priority disease categories at household level.

**DC**	**Financial/income loss**					**Mortality**					**School**					**Productivity**				
	**β (ref)**	**Std. error**	**e^**β**^ (OR)**	**95% Confidence interval for e**^****β****^		**β (ref)**	**Std.** **error**	**e^**β**^ (OR)**	**95% Confidence interval for e**^****β****^		**β (ref)**	**Std.** **error**	**e^**β**^ (OR)**	**95% Confidence interval for e**^****β****^		**β (ref)**	**Std.** **error**	**e^**β**^ (OR)**	**95% Confidence interval for e**^****β****^	
R	1.22 (O)	0.537	3.39^*^	1.184	9.727															
N						−1.46 (R)	0.381	0.23^***^	0.111	0.492	1.25 (R)	0.628	3.49	1.022	11.976					
S	1.71 (O)	0.639	5.55^**^	1.585	19.432	−0.75 (R)	0.374	0.47^*^	0.227	0.984						1.00 (N)	0.470	2.72^*^	1.084	6.841
G	1.109 (O)	0.557	3.03^*^	1.018	9.030	−1.19 (R)	0.286	0.30^***^	0.172	0.528						0.89 (N)	0.390	2.45^*^	1.140	5.266
E	−1.65 (S)	0.707	0.19^*^	0.048	0.767	−2.91 (O)	1.000	0.05^**^	0.008	0.386										
						−3.36 (R)	0.873	0.04^***^	0.006	0.192										
						−1.91 (N)	0.915	0.15^*^	0.025	0.895										
						−2.61 (S)	0.903	0.07^**^	0.013	0.430										
						−2.16 (G)	0.870	0.12^*^	0.021	0.634										
Y	1.82 (O)	0.741	6.17^*^	1.443	26.380	1.05 (N)	0.502	2.85^*^	1.066	7.630						−2.00 (O)	0.705	0.14^**^	0.034	0.537
	1.76 (E)	0.791	5.79^*^	1.228	27.322	0.791 (G)	0.384	2.21^*^	1.040	4.682						−1.44 (R)	0.450	0.24^***^	0.098	0.571
						2.95 (E)	0.917	19.17^***^	3.177	115.638						−1.76 (S)	0.504	0.17^***^	0.062	0.446
																−1.69 (G)	0.452	0.19^***^	0.076	0.448
																−1.66 (E)	0.799	0.19^*^	0.040	0.915

*R, respiratory diseases; N, neurological diseases; S, skin diseases; G, gastrointestinal diseases; E, ectoparasites; Y, systemic diseases; O others*.

*** The parameters β were significant at 0.001 level;

** at 0.01;

* *at 0.05*.

*The disease impacts were compared to each of the seven disease categories (each considered as a reference category in turn) in a series of analyses to determine the statistical significance of the differences*.

**Table 7:** Continued

**DC**	**Time it took to treat animals**					**Cost**					**Value**					**Malnutrition**				
	**β (ref)**	**Std.** **Error**	**e^**β**^ (OR)**	**95% Confidence interval for e**^****β****^		**β (ref)**	**Std.** **Error**	**e^**β**^ (OR)**	**95% Confidence interval for e**^****β****^		**β (ref)**	**Std.** **Error**	**e^**β**^ (OR)**	**95% Confidence interval for e**^****β****^		**β (ref)**	**Std.** **Error**	**e^**β**^ (OR)**	**95% Confidence interval for e**^****β****^	
R	−1.75 (O)	0.573	0.17^**^	0.056	0.534															
N	−3.16 (O)	0.753	0.04^***^	0.010	0.185	−1.44 (O)	0.718	0.24^***^	0.058	0.963										
	−1.41 (R)	0.552	0.244^*^	0.083	0.719	−1.62 (R)	0.468	0.19^***^	0.079	0.493										
S	−1.50 (O)	0.681	0.22^*^	0.059	0.845	1.58 (N)	0.599	4.84^**^	1.496	15.647										
	1.659 (N)	0.683	5.26^*^	1.378	20.050															
G	−1.72 (O)	0.599	0.18^**^	0.056	0.583	1.34 (N)	0.505	3.82^**^	1.423	10.281						1.60 (N)	0.613	4.97^**^	1.496	16.529
	1.45 (N)	0.586	4.25^*^	1.35	13.403															
E	−2.29 (O)	0.961	0.10^*^	0.015	0.660											2.16 (N)	1.022	8.63^*^	1.164	63.922
Y	−2.54 (O)	0.831	0.08^**^	0.015	0.402						−1.29 (R)	0.553	0.28^*^	0.093	0.816					
											−1.32 (S)	0.617	0.27^*^	0.080	0.896					

*R, respiratory diseases; N, neurological diseases; S, skin diseases; G, gastrointestinal diseases; E, ectoparasites; Y, systemic diseases; O, others*.

*** The parameters β were significant at 0.001 level;

** at 0.01;

* at 0.05

*The disease impacts were compared to each of the seven disease categories (each considered as a reference category in turn) in a series of analyses to determine the statistical significance*.

**Table 7:** Continued

**DC**	**Psychological**					**Human health**					**Agroecology and production system**				
	**β (ref)**	**Std. Error**	**e^**β**^ (OR)**	**95% Confidence interval for e**^****β****^		**β (ref)**	**Std. Error**	**e^**β**^ (OR)**	**95% Confidence interval for e**^****β****^		**β (ref)**	**Std. Error**	**e^**β**^ (OR)**	**95% Confidence interval for e**^****β****^	
N						−2.29 (R)	1.067	0.10^*^	0.012	0.814					
S											LMC 4.43 (R)	1.255	83.84^***^	7.17	980.43
											MMC 3.87 (N)	1.576	47.84^*^	2.18	1049.62
											MPA^∧^				
G	−1.24 (R)	0.579	0.29^*^	0.09	0.90	−1.32 (R)	0.604	0.27^*^	0.082	0.870					
	−1.67 (N)	0.656	0.19^*^	0.05	0.68										
E															
Y	1.91(S)	0.932	6.78^*^	1.09	42.13	2.93 (N)	1.145	18.64^*^	1.97	175.73	HMC −2.06 (R)	0.70	0.13^**^	0.03	0.50
	1.84 (G)	0.709	6.31^**^	1.57	25.30	2.34 (S)	1.075		1.26		LMC 1.805 (R)	0.867	6.08^*^	1.11	33.24
						1.95 (G)	0.663	10.3^*^	1.92	85.36	HMC −2.93 (S)	1.243	0.05^*^	0.01	0.61
								7.03^**^		25.76	LMC −2.62 (S)	1.318	0.07^*^	0.01	0.96
											MMC −4.16 (S)	1.580	0.02^**^	0.001	0.35
											MPA^∧^				

*R, respiratory diseases; N, neurological diseases; S, skin diseases; G, gastrointestinal diseases; E, ectoparasites; Y, systemic diseases; O, others*.

*** The parameters β were significant at 0.001 level;

** at 0.01;

* at 0.05

*The disease impacts were compared to each of the seven disease categories (each considered as a reference category in turn) in a series of analyses to determine the statistical significance*.

### Disease Knowledge of Farmers

**Figure 2:**
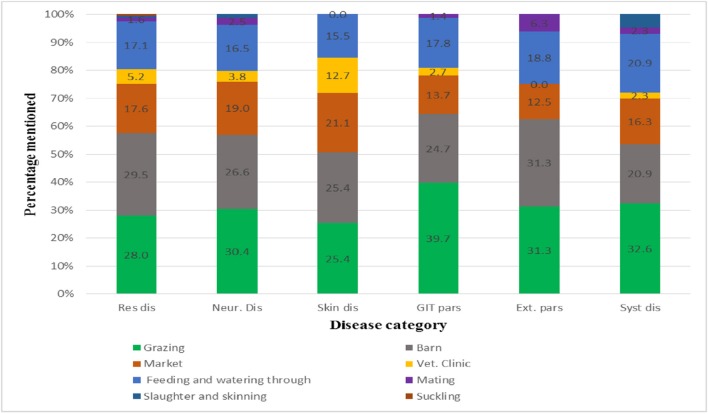
Major disease transmission pathways identified by farmers.

The FGDs found that, in general, farmers have a fair amount of knowledge about disease transmission pathways between animals. Identified common transmission pathways or situations leading to transmission of diseases among animals were feeding and watering troughs, common barns, sharing grazing areas, communal markets, slaughter and skinning places, and suckling (Figure 2).

Farmers said transmission of respiratory diseases was mainly related to inadequate ventilation in barns, poor sanitation, common grazing areas, market places and during use of communal feeding and watering troughs. Farmers were also knowledgeable about predisposing stress factors associated with housing, handling and marketing that can cause clinical presentation of pasteurellosis (25, 26). Farmers clearly stated that GIT parasites are mostly transmitted during grazing.

The household survey explicitly prompted households to acquire information on zoonotic diseases. Forty-six percent of the respondents said they knew about zoonotic diseases, but only 36% were actually able to name and describe a zoonotic disease. Anthrax, rabies, bovine tuberculosis and taeniasis were mentioned by 19.9, 7.6, 1.9, and 2.5% of respondents, respectively, while 1.4% of respondents named both anthrax and rabies. Anthrax was mostly mentioned in households in lowland agroecologies. There was no significant difference on knowledge about zoonoses between gender and age groups. Overall, both men and women farmers had low awareness of zoonotic diseases.

**Figure 3:**
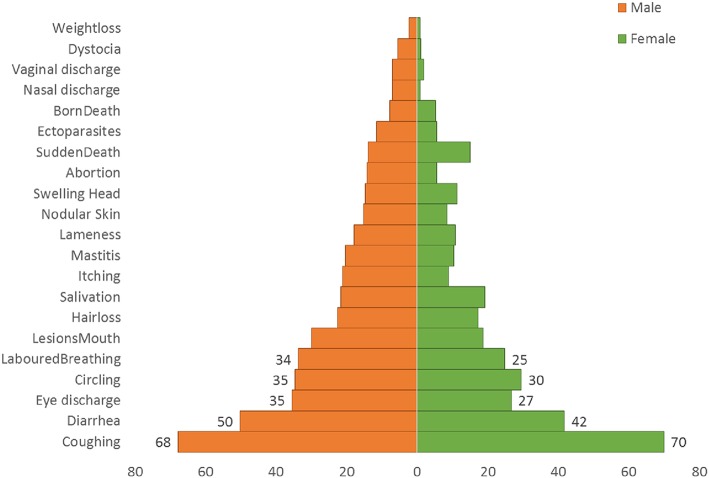
Frequency percentage of clinical signs mentioned by gender.

**Figure 4:**
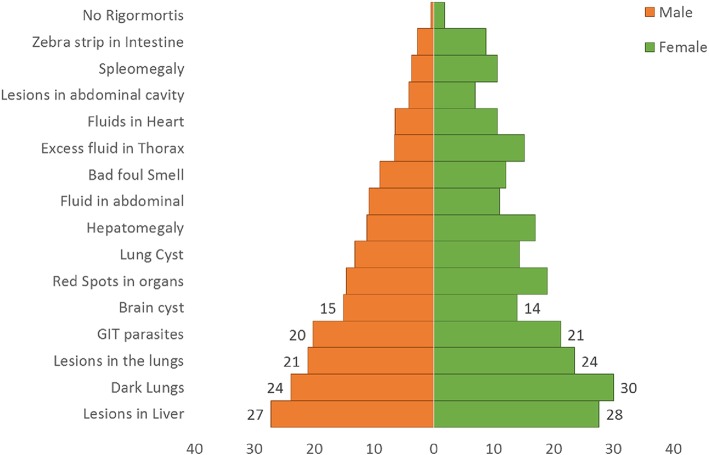
Frequency percentage of postmortem signs mentioned by gender.

Regarding the knowledge of men and women farmers about small ruminant diseases, the household survey revealed that despite the differences in household roles, both men and women unvaryingly described the clinical signs in live animals similarly (Figure 3) and reported similar observations of disease in carcasses of slaughtered animals (Figure 4).

### Disease Coping Mechanism or Strategy

The FGDs investigated disease coping strategies. These included mechanisms to prevent and control diseases, combined with strategies to reduce economic loss and ensure survival at difficult times. The use of modern veterinary services included vaccination and treatment with modern drugs. Traditional treatment and beliefs relied on traditional remedies and treatment practices. Health management activities covered isolation of sick animals, grazing management, keeping barns clean, proper carcass disposal of dead animals, proper supplementary feeding, and prevention from heat stress.

**Figure 5:**
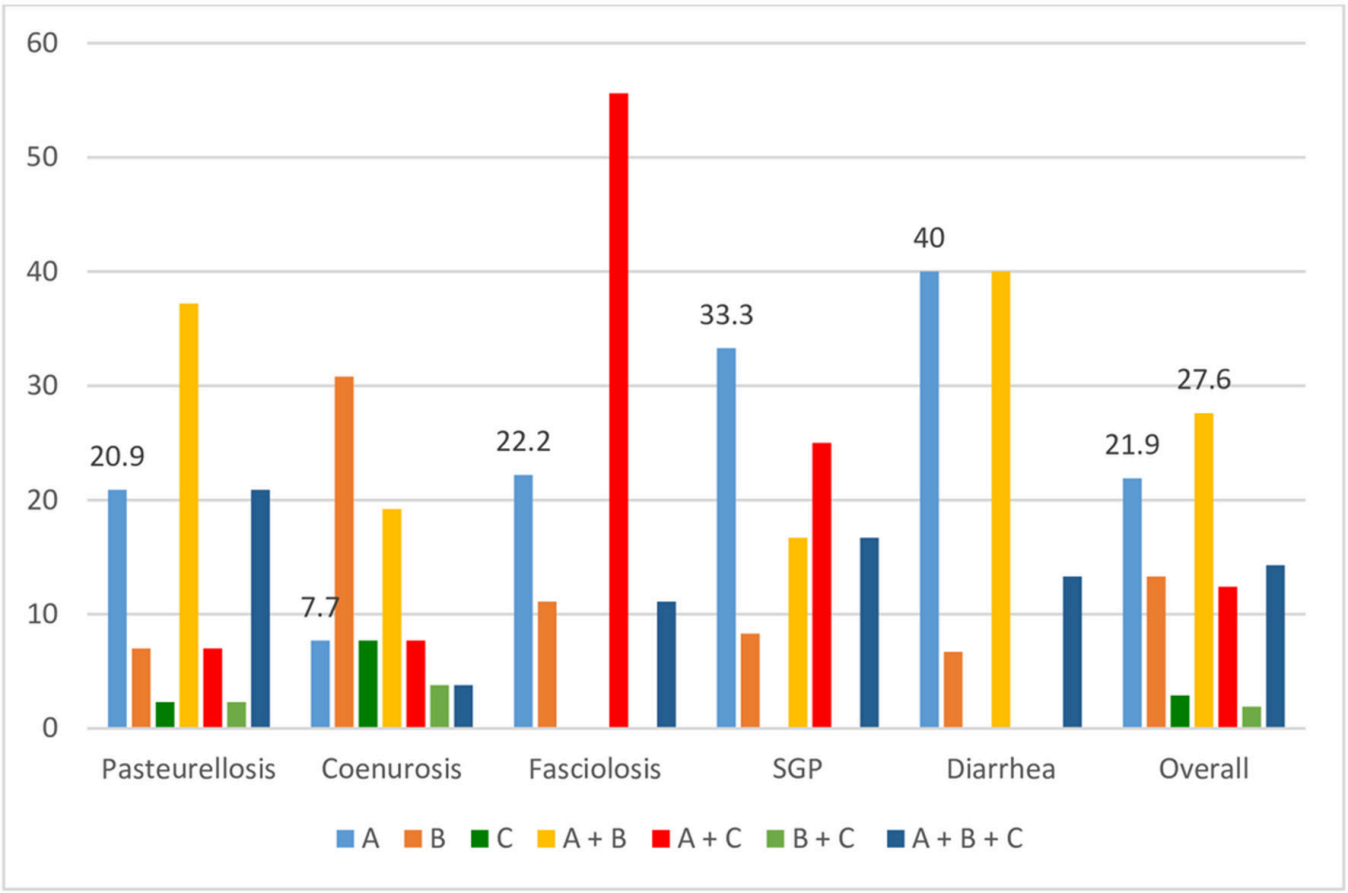
Percentage of respondents (FGD) using different coping mechanisms against the five top small ruminant disease (A, use modern veterinary service; B, traditional treatment and beliefs; C, health management activities. +indicates combination).

Most of the farmers (27.6%) often depend on both modern veterinary medicine and traditional treatment and beliefs for prevention and control of small ruminant diseases. However, only a few farmers seem to use other health management activities (Figure 5).

From the focus groups, 13% reported traditional treatment practices like drenching with herbal preparations of onion (*Allium cepa*), ginger (*Zingiber officinale*), tobacco leaf (*Nicotiana tabacum*), black cumin (*Nigella sativa*), “Feto” (Lepidium sativum L.); fumigation with “kebericho” (Echinops kebericho) through nose and mouth of sick animals; and using holy water for treatment and control of pasteurellosis. For treatment of coenurosis, remedies like drenching a local alcoholic drink and tobacco leaf juice through the nose, hot iron branding of the forehead or immersing the infected sheep in well water and fumigating with burned plastic were mentioned. For treatment of diarrhea, drenching of herbal preparations made of ginger (*Zingiber officinale*) and “Feto” (*Lepidium sativum* L.) and mineral soil locally called “Bole” were used. Topical applications or dressing lesions with honey, butter, pepper powder and salty water were practiced to treat sheep and goat pox.

The strategies to reduce economic loss during vulnerable times included sale of animals at lower price, slaughter of animals for home consumption, contractually own (rent) flock from other people/relatives, shifting to other business activities, buying new flock by selling cattle or crop, and searching for financial support from relatives/government/NGOs.

## Discussions

This study identified disease priorities as perceived by farmers and provided insights into why the diseases were considered important. The findings show that priorities of national disease control programs do not fully match priorities of farmers. The study used mixed research methods combining FGDs and household survey. This approach provided a better understanding of disease priorities than either approach alone could. The FGDs helped understand the nature of the data and the meaning of what participants said, which vary across the study area and across groups of smallholders. This influenced data analysis and interpretation. The household survey allowed for a deeper exploration of the qualitative data from FGDs to build a fuller picture of the impact of diseases, the actions taken by smallholders when facing small ruminant diseases and insights into possible reasons for these actions.

The Government of Ethiopia recognized the importance of livestock health as a priority in the Ethiopian livestock master plan (LMP) (27). The LMP considers Peste des petits ruminants (PPR), sheep and goat pox (SGP), and CCPP as important small ruminant diseases based on their impact on rural households and their livelihoods, intensification pathways and implications for international trade.

In this study, livestock owners listed several typical multifactorial production diseases as priorities, such as pasteurellosis, coenurosis, and GIT parasites. However, these diseases are largely neglected in disease control efforts. Similarly, Gari et al. (28) documented sheep and goat diseases in two districts of Afar regional state representing the lowland pastoral and agro-pastoral production systems and found respiratory syndrome/CCPP, sheep and goat pox, diarrhea and tick- and tick-borne diseases as highly ranked health problems. They also described lung worms, pneumonia and septicemia pasteurellosis as the most suspected respiratory diseases and called for conventional surveillance in the future.

This study emphasizes a disparity between community priorities and government priorities. Likewise, it is widely agreed that national and global surveillance systems should focus on transmissible diseases affecting international trade or have importance from a public health point of view. However, production diseases are often not perceived to be a serious enough threat to gain attention. Not surprisingly, inaccurate disease surveillance reports are common, and even if targeted by national surveillance systems, disease occurrence is likely to be underreported. Being off the radar of any surveillance programs also means that there is no other investments in support of prevention and control of these diseases. Hence our study highlights the need for control programs and access to veterinary inputs that meet needs of smallholders.

Impact of the diseases presented here can be reduced by proper health management at herd or community level. But this study also found that such prevention and control measures are rarely implemented and farmers rely on veterinary inputs or traditional treatments. This is clearly an area where improved capacity of farmers is needed, which can be achieved by adequate extension systems or other animal health service provision that support and facilitate prevention. Farmers mentioned a range of coping mechanisms and/or strategies against small ruminant diseases. Traditional practices play a big role in these coping mechanisms. While their positive impact has been previously described and can be used to provide economical solutions to improve productivity of animals and reduction in poverty of the poor farmers (29), traditional methods are not always the best or most effective for treating infectious diseases. What these practices reflect though, is the need and willingness of farmers to do something in response to disease occurrence.

Catley and Mohammed (30), described livestock disease scoring method and used “importance indicators” and “difference indicators” for pair-wise comparisons to understand what herders thought were the most important livestock diseases to cause economic and production losses. In this study, livestock owners provided a range of specific reasons for ranking diseases. The reasons included the resulting economic and production loss (high mortality, high morbidity, market price devaluation, inedible meat, unmarketable skin, no treatment, and recovery); characteristics of the diseases (high transmission rate, acute and fatal); and occurrence (frequent occurrence or endemicity).

Farmers attributed high mortality to systemic diseases like PPR and anthrax, respiratory diseases like pasteurellosis and CCPP and GIT diseases caused by liver fluke. Their reasoning for priority was coherent with knowledge on how these diseases affect infected animals (see the specific diseases mentioned on Manual of Diagnostic Tests and Vaccines for Terrestrial Animals, 2019).

Farmers mentioned lack of available treatment as the main reason to prioritize neurological diseases such as coenurosis. Achenef et al. (31) also declared that no single satisfactory treatment method has been devised under field conditions, although trephining has been advocated for coenurosis. Dogs, which are part of the coenurosis transmission cycle, play an important role for herders in pastoral and agro-pastoral areas and may explain the high incidence, as the presence of freely roaming dogs on grazing land greatly contributes to the existence of the disease (32).

Catley et al. (3) indicated gender analysis tools that can be tailored to animal health activities. These include livestock keeping household activity profiling; livestock activity, access to and control over resources profiling; livestock resources and benefits index; and a practical and strategic gender needs in livestock management index.

In the design of this study, we paid great attention to capture the views of men and women by conducting separate FGDs and by ensuring women-headed households were included in the household survey. There were no major differences in men and women households and focus groups in identifying and scoring priority disease constraints. Similarly, the knowledge of men and women farmers were similar in describing the clinical signs in live animals, as well as their observation in slaughtered animals. The findings of Galie et al. (33) also showed that both women and men were involved in cattle health management in Tanzania and had similar knowledge of diseases. With the participant selection, there might have been introduction of potential “elite bias” (34) in our study. The traditional healers included in the men FGD tended to be the most knowledgeable and articulate members of the group, which may have affected comparison of men and women FGDs. However, the primary objective of the study to identify the major small ruminant disease would not be affected, and any bias introduced through the non-random selection of participants seemed minimal.

The study also found that farmers have good knowledge about disease transmission pathways between animals but that both men and women farmers had low awareness of zoonotic diseases. In this regard, it is important to note that none of the focus groups mentioned impact of zoonotic diseases on human health as reason for prioritizing diseases. However, considering the high prevalence of zoonoses and the close interactions of people and animals in these production systems, their importance cannot be overstated. Kinati et al. (35) provided insights into the role of division of labor related to small ruminant health management in the same cohort targeted in our study and showed different levels of contact/involvement of different household members in possible transmission pathways. This is clearly another issue that should be addressed through advisory and extension services by involving both men and women.

## Data Availability Statement

The datasets generated for this study are available on request to the corresponding author.

## Ethics Statement

The studies involving human participants were reviewed and approved by ILRI Institutional Research Ethics Committee (ILRI IREC), ILRI IREC is accredited by the National Commission for Science, Technology and Innovation (NACOSTI) in Kenya. The patients/participants provided their written informed consent to participate in this study.

## Author Contributions

BW, BA, HD, AM, and WK conceived and designed the study and followed up and monitored data collection. BA analyzed the data. BA, BW, and SG conceptualized and drafted the paper. All authors read, commented, and approved the final manuscript.

### Conflict of Interest

The authors declare that the research was conducted in the absence of any commercial or financial relationships that could be construed as a potential conflict of interest.
